# From Batch to Continuous Flow Bioprocessing: Use of an Immobilized γ-Glutamyl Transferase from *B. subtilis* for the Synthesis of Biologically Active Peptide Derivatives

**DOI:** 10.1021/acs.jafc.2c03702

**Published:** 2022-09-23

**Authors:** Marina S. Robescu, Francesca Annunziata, Valeria Somma, Cinzia Calvio, Carlo F. Morelli, Giovanna Speranza, Lucia Tamborini, Daniela Ubiali, Andrea Pinto, Teodora Bavaro

**Affiliations:** † Department of Drug Sciences, University of Pavia, viale Taramelli 12, Pavia 27100, Italy; ‡ Department of Pharmaceutical Sciences, University of Milano, via Mangiagalli 25, Milano 20122, Italy; § Department of Chemistry, University of Milano, via Golgi 19, Milano 20122, Italy; ∥ Department of Biology and Biotechnology “L. Spallanzani”, University of Pavia, via Ferrata 1, Pavia 27100, Italy; ⊥ Department of Food, Environmental and Nutritional Sciences, University of Milano, via Celoria 2, Milano 20133, Italy

**Keywords:** γ-glutamyl transferase, *Bacillus subtilis*, *kokumi* peptides, enzyme immobilization, flow chemistry

## Abstract

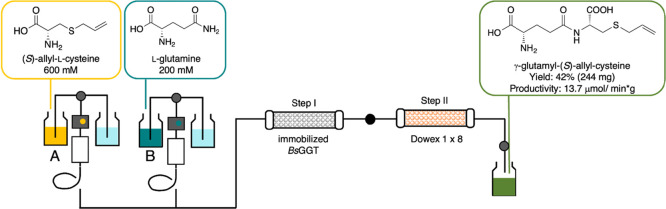

γ-Glutamyl-peptides are frequently endowed with biological activities. In this work, “*kokumi* peptides” such as γ-glutamyl-methionine (**1**) and γ-glutamyl-(*S*)-allyl-cysteine (**2**), as well as the neuroprotective γ-glutamyl-taurine (**3**) and the antioxidant ophthalmic acid (**4**), were synthesized through an enzymatic transpeptidation reaction catalyzed by the γ-glutamyl transferase from *Bacillus subtilis* (*Bs*GGT) using glutamine as the γ-glutamyl donor. *Bs*GGT was covalently immobilized on glyoxyl-agarose resulting in high protein immobilization yield and activity recovery (>95%). Compounds **1**–**4** were obtained in moderate yields (19–40%, 5–10 g/L) with a variable purity depending on the presence of the main byproduct (γ-glutamyl-glutamine, 0–16%). To achieve process intensification and better control of side reactions, the synthesis of **2** was moved from batch to continuous flow. The specific productivity was 1.5 times higher than that in batch synthesis (13.7 μmol/min*g), but it was not accompanied by a paralleled improvement of the impurity profile.

## Introduction

γ-Glutamyl-peptides (γ-E-peptides) have been reported to exhibit a plethora of bioactivities,^1^ including anti-inflammatory (e.g., γ-glutamylvaline, γ-E-Val), neuroprotective (e.g., γ-glutamyl-β-amino-ethan-sulfonic acid or γ-glutamyltaurine, γ-E-Tau),^2^ antioxidant (e.g., ophthalmic acid or γ-glutamyl-l-2-aminobutyrylglycine, γ-E-Abu-Gly), and appetite-suppressing (e.g., γ-glutamyltriptophan, γ-E-Trp) effects as well as *kokumi* taste (e.g., γ-glutamylphenylalanine, γ-E-Phe, γ-glutamylmethionine, γ-E-Met, γ-glutamyl-(*S*)-allyl-cysteine, γ-E-(*S*)-allyl-Cys, and γ-glutamylleucine, γ-E-Leu).^3^*Kokumi* is a gustative sensation characterized by roundness, mouthfulness, and continuity. *Kokumi*-active peptides are naturally occurring flavor enhancers which induce a rich and long-lasting mouthfeel of food by interacting with the calcium sensor receptor (CaSR).^3^

γ-Glutamyl transferases (GGTs, EC 2.3.2.x) catalyze the cleavage of the γ-glutamyl bond of γ-glutamyl compounds such as glutamine (Gln) and glutathione and the transfer of the γ-glutamyl moiety to amino acids and peptides. GGTs are found in mammals, fungi, bacteria, and plants.^4,5^ GGTs from bacterial sources are generally preferred as biocatalysts for preparative purposes. They are easily produced and purified, minimizing the possibility of viral contamination, a common risk associated with the use of enzymes from mammalian sources. In addition, the use of glutamine as a γ-glutamyl donor substrate instead of glutathione, which is required by GGTs from higher organisms, can reduce significantly the costs of raw materials. Specifically, GGTs from *Bacillus subtilis sp*. raise a considerable interest as biocatalysts for the synthesis of γ-E-derivatives for human consumption owing to their Generally Recognized As Safe status.^6^

**Scheme 1:**
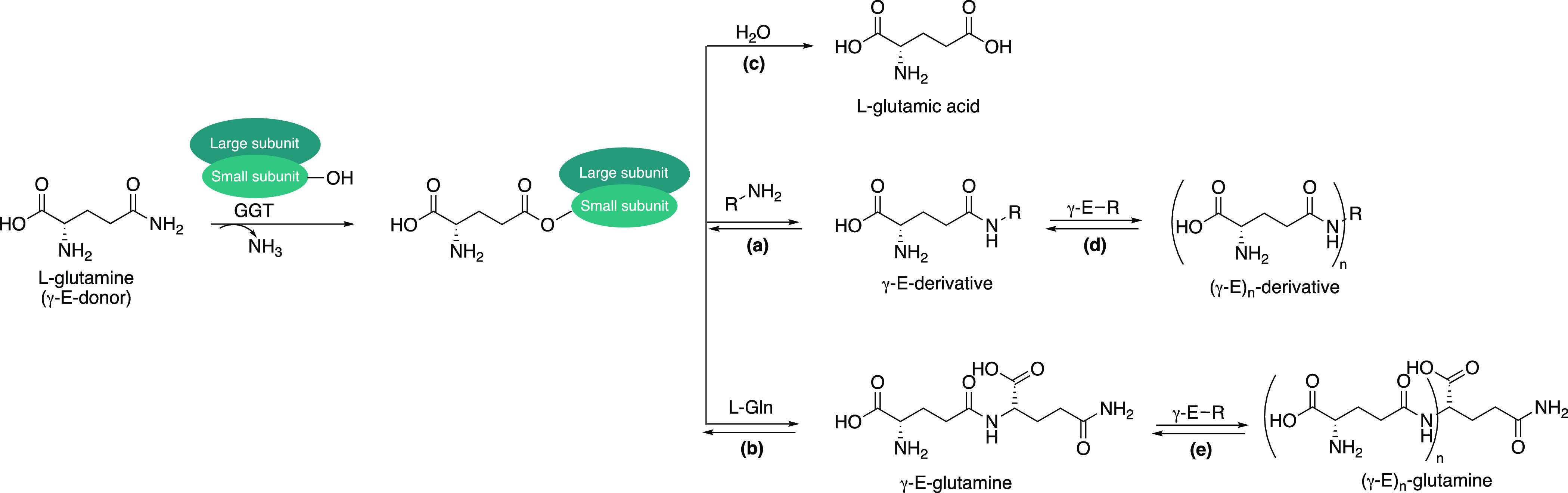
Reactions Catalyzed by γ-Glutamyl Transferases (GGTs): (a) Transpeptidation; (b) Autotranspeptidation; (c) Hydrolysis; (d) and (e) Polyglutamylation of γ-E-Derivative and γ-E-Glutamine, Respectively. γ-E-R = γ-Glutamyl Compounds Formed During the Reaction

Despite the potential of GGTs for the synthesis of γ-E-derivatives, their use as biocatalysts for preparative purposes is plagued by some drawbacks. In fact, the transpeptidation reaction (Scheme 1, path a) is accompanied by autotranspeptidation (Scheme 1, path b) and hydrolysis (Scheme 1, path c) of the donor substrate (Gln), thus leading to γ-E-Gln and glutamic acid, respectively. Besides hydrolysis and autotranspeptidation, GGTs from *Bacillus spp*. can also catalyze the formation of polyglutamylated compounds (Scheme 1, paths d and e):^6^ as the concentration of γ-E-Gln and γ-E-derivative increases, indeed, these products can compete as γ-glutamyl acceptors, thus generating (poly)-γ-E-derivatives. Furthermore, beyond the hydrolysis reaction which is irreversible, all the glutamylated species formed during the reaction (i.e., γ-E-derivative, γ-E-Gln, and their polyglutamylated species) can enter again in the catalytic cycle acting as γ-glutamyl donors, thus releasing their γ-glutamyl moiety. *Bacillus sp*. GGTs have been proposed as biocatalysts for the preparative synthesis of γ-glutamyl derivatives;^7−13^ nevertheless, all these side reactions reduce the product yield and increase the complexity of the reaction mixture, thereby hampering product purification.

In order to maximize the formation of transpeptidation products, mutagenesis experiments were carried out to improve the catalytic efficiency of GGTs as biocatalysts according to path a (Scheme 1). To this purpose, a few variants of GGTs were reported.^14−16^*Bacillus licheniformis* ER15 GGT (*Bl*GGT) was engineered in an attempt of minimizing the autotranspeptidation reaction. The Arg109Lys mutation increased transpeptidation activity and catalytic efficiency in the synthesis of l-theanine (80% vs 60% conversion with the wild-type enzyme, respectively, under the same reaction conditions).^14^ Later on, the transpeptidase activity of a mutant GGT, obtained by inserting the lid-loop from *Escherichia coli* GGT (*Ec*GGT) in the structure of *Bacillus subtilis* GGT (*Bs*GGT), naturally lacking it, was investigated.^15^ The mutant GGT showed enhanced transpeptidase activity with respect to the wild-type enzyme. However, the mutant enzyme maintained the ability of the wild-type parent GGT to catalyze the formation of polyglutamylated derivatives, thus hampering its application in preparative syntheses.^15^ The transpeptidase activity of GGTs can be also selectively enhanced by a fine tuning of the reaction conditions (donor/acceptor ratio, pH, time, temperature, and enzyme amount), even assisted by advanced reaction and enzyme engineering tools based on flow biocatalysis and enzyme immobilization, respectively. On the one hand, flow biotransformations combine flow chemistry’s throughput to the catalytic power of enzymes, thus providing new solutions to achieve high productivity, better process control, and less waste. On the other hand, immobilized enzymes used in flow systems are far more stable than the native (nonimmobilized) enzymes and can be recovered and reused for repeated reactions.^17^

In the case of GGT-catalyzed biotransformations, which are plagued by many concurrent reactions (Scheme 1), a flow set-up might assist in finely tuning the substrate/product time of contact with the biocatalyst, thus allowing to track and mitigate the side reactions (i.e., autotranspeptidation and hydrolysis). Furthermore, continuous removal of the product from the reaction site by using an in-line purification set-up could be an option to reduce poly-γ-E-derivative formation. In parallel, covalent immobilization of GGTs can provide an active, stable, and reusable biocatalyst.

**Figure 1:**
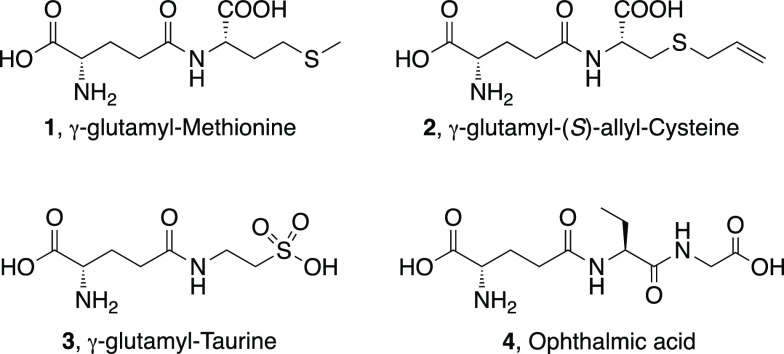
γ-E-peptides synthesized in this work.

In this paper, the batch synthesis of biologically active γ-E-peptides such as γ-E-Met (**1**), γ-E-(*S*)-allyl-Cys (**2**), γ-E-Tau (**3**), and ophthalmic acid (**4**) in a fully aqueous medium catalyzed by immobilized GGTs from *Bacillus subtilis* (*Bs*GGT) was carried out (Figure 1). Covalent immobilization of *Bs*GGT and flow chemistry principles were jointly exploited for the first time, indeed, in the synthesis of γ-E-(*S*)-allyl-Cys (**2**), a *kokumi* peptide, with the aim both to minimize side reactions and to improve the turnover and productivity of the biocatalyst for process intensification. An in-line purification step was envisaged to avoid tedious separation techniques as applied in the batch reactions.

## Materials and Methods

### Materials

l-Glutamic acid γ-(4-nitroanilide) (G*p*NA), glycylglycine (Gly-Gly), 4-nitroaniline, l-serine, l-glutamine, l-methionine, Bradford reagents, glycidol, sodium periodate (NaIO_4_), sodium borohydride (NaBH_4_), ethylenediamine (EDA), glutaraldehyde (GA), taurine, potassium phosphate, 1-fluoro-2,4-dinitrobenzene (Sanger’s reagent), and high-pressure liquid chromatography (HPLC)-grade solvents were purchased from Sigma Aldrich (Milano, Italy). l-2-Aminobutyrylglycine (Abu-Gly) was from Bachem (Bubendorf, Switzerland). (*S*)-Allyl-cysteine was prepared as previously reported.^18^ Sepharose CL-6B (agarose; AG) was from GE Healthcare (Uppsala, Sweden). Sepabeads RelyZyme EP112/S was a gift of Resindion S.r.l (Binasco, Italy). Sodium bicarbonate was from Carlo Erba (Cornaredo, Italy).

Dowex 1 × 8 resin (200–400 mesh, chloride form) was purchased from Sigma Aldrich (Milano, Italy). The resin was used in acetate form. Before use, the resin (15 g) was suspended in 50 mL of water, filtered, and washed with water until a clear solution was obtained. Then, the resin was conditioned in acetate form by washing with (1) 50 mL of 1 M NaOH, (2) 25 mL of water, (3) 50 mL of 2 M AcOH, and (4) 50 mL of 0.5 M AcOH. The resin was stored at 4 °C in 0.5 M AcOH until use.

Spectrophotometric assays were performed by using a Shimadzu UV-1601 UV–visible spectrophotometer equipped with magnetic stirring. Analytical thin-layer chromatography (TLC) was performed on silica gel F_254_ precoated aluminum sheets (0.2 mm layer) (Merck, Darmstadt, Germany); the mobile phase was *iso*-propanol/acetone/water/AcOH (35:35:23:7). Analyte detection was performed by using 1% w/v ninhydrin in ethanol, followed by heating at 150 °C ca. HPLC analyses were carried out with a Jasco LC-400 instrument equipped with a Jasco UV-4070 UV/vis detector, by using a 250 × 4.6 mm Gemini RP C18 column (Phenomenex, Torrance, CA, USA). ^1^H-NMR spectra were acquired at 400.13 MHz using a Bruker Advance 400 spectrometer (Bruker, Karlsruhe, Germany) interfaced with a workstation running *Windows* operating system and equipped with a TOPSPIN software package. Chemical shifts (δ) are given in ppm and are referenced to the solvent’s signal (δ_D2O_ = 4.79 ppm). Electrospray ionization mass spectrometry (ESI-MS) spectra were acquired using a Thermo Finnigan LCQ Advantage spectrometer interfaced with a computer running Xcalibur software package (Thermo Fisher Scientific, Waltham, MA, USA). Continuous flow biotransformations were performed using a R2^+^/R4 flow reactor equipped with an Omnifit glass column (6.6 mm i.d. × 150 mm length). The temperature sensor sits on the wall of the reactors. Pressure was controlled by using back-pressure regulators. The *N*-terminal His-tagged GGT from *Bacillus subtilis* 168 (*Bs*GGT) was produced as previously described by Morelli et al.^6^

### Methods

#### *Bs*GGT Activity Assay

The standard activity assay was performed at room temperature in 100 mM Tris–HCl buffer pH 8.5 containing 1 mM G*p*NA, 100 mM Gly-Gly, and an appropriate amount of enzyme (free *Bs*GGT: 13 μg; immobilized *Bs*GGT: 5–10 mg, under magnetic stirring) in a final volume of 2 mL. The reaction was monitored spectrophotometrically by measuring the formation of 4-nitroaniline at 410 nm in the kinetic mode. The amount of 4-nitroaniline produced by the enzyme was quantified by using a calibration curve and an extinction coefficient of 8.3 mM^–1^ cm^–1^. One unit of *Bs*GGT was defined as the amount of enzyme that produces 1 μmole of 4-nitroaniline per minute from G*p*NA in the presence of the acceptor Gly-Gly.

#### Preparation of Agarose-Based Carriers

Glyoxyl-agarose (GLX-AG) was prepared as previously reported.^19^ Briefly, 5.0 g of agarose was suspended in a solution of 1.4 mL of deionized water and 2.4 mL of 1.7 M NaOH containing 14.2 mg/mL NaBH_4_. Subsequently, 1.7 mL of glycidol was added dropwise keeping the vessel at 4 °C in an ice bath. After the addition of glycidol, the reaction was kept under gentle stirring overnight at room temperature. The suspension was filtered, and the carrier was washed abundantly with deionized water. Oxidation was initiated by adding 34 mL of 100 mM NaIO_4_. The reaction was carried out for 2 h at room temperature, and then, the carrier was filtered under reduced pressure, washed abundantly with deionized water, and stored at 4 °C until use.

Glutaraldehyde-activated agarose (GA-EDA-AG) was prepared as described in the literature.^20^ GLX-AG (1.0 g) was aminated using 12.9 mL of 2 M EDA at pH 10.0 for 2 h and subsequently reduced for 2 h with 14 mg of NaBH_4_. The suspension was filtered, and the carrier was washed abundantly with deionized water. Subsequently, the EDA-activated agarose was suspended in 1.1 mL of 200 mM phosphate buffer pH 7.0, and 1.7 mL of a solution of GA (25% v/v) was added. The mixture was kept under mechanical stirring for 16 h at room temperature in the darkness. The activated carrier was washed abundantly with deionized water and stored at 4 °C until use.

#### Preparation of ReliZyme EP112/S-Based Carriers

Commercial ReliZyme EP112/S was hydrated with distilled water for 1 h under mechanical stirring at room temperature. The acrylic resin was then filtered under vacuum and activated with different chemical groups as follows.

Glyoxyl-ReliZyme EP112/S (GLX-ReliZyme) was prepared according to a procedure previously reported.^21^ ReliZyme EP112/S (1.0 g) was subjected to epoxide ring opening in 13 mL of 0.5 M sulfuric acid for 2 h at room temperature. Then, the activated carrier was filtered under reduced pressure and washed with distilled water until a neutral pH was observed. The diol groups of the carrier were further oxidized with 4 mL of 100 mM NaIO_4_ for 2 h at room temperature. Finally, the glyoxyl-activated carrier was washed with distilled water and stored at 4 °C until use.

Glutaraldehyde-activated ReliZyme EP112/S (GA-EDA-ReliZyme) was prepared as reported in the literature.^20^ The aldehyde support GLX-ReliZyme (1.0 g) was suspended in 12.9 mL of 2 M EDA pH 10.0 and kept at room temperature under mechanical stirring for 2 h. Then, 14 mg of NaBH_4_ was added, and the reaction mixture was maintained under stirring for additional 2 h. The carrier was then filtered under reduced pressure on a glass filter, washed with deionized water, and used for the following activation with GA. The aminated carrier (1.0 g) was suspended in 1.1 mL of 200 mM phosphate buffer pH 7.0, and then, 1.7 mL of GA (25% v/v) was added. The mixture was kept under mechanical stirring for 16 h at room temperature in the dark. The resin was filtered, washed with deionized water, and stored at 4 °C until use.

Glucosamine-activated ReliZyme EP112/S (GlcN-ReliZyme) was prepared as reported in the literature.^22^ The epoxy-activated ReliZyme EP112/S (1.0 g) was suspended in 12.8 mL of 1 M glucosamine. The pH of the resulting solution was adjusted to 7.5 or 10.0 by adding diluted NaOH, and the suspension was kept under mechanical stirring at room temperature for 24 h; then, the activated carrier was washed with deionized water. The resultant resin was oxidized with 27 mL of 15 mM NaIO_4_ for 1 h at room temperature. The resin was filtered, washed with deionized water, and stored at 4 °C until use.

#### *Bs*GGT Immobilization

For all the immobilization procedures, an enzyme loading of 1 mg per gram of carrier and a 10:1 ratio volume of immobilization reaction/volume of the carrier were used. During immobilization, the supernatant was monitored by measuring the amount of protein in solution (Bradford assay) and the residual activity of the supernatant was checked by the standard activity assay described above.

Immobilization of *Bs*GGT on GLX-AG was performed by following a standard protocol.^23^ Briefly, GLX-AG was washed abundantly with 50 mM NaHCO_3_ buffer pH 10.0 and then filtered under reduced pressure until dryness. Then, 77 μL of *Bs*GGT (6.5 mg/mL) was solubilized in 6.4 mL of NaHCO_3_ buffer. Then, 500 mg of carrier was added, and the suspension was allowed to stir for 3 h at room temperature. Finally, 7 mg of NaBH_4_ was added to the mixture and incubated for 30 min for imine reduction. The immobilized enzyme was then filtered, washed with deionized water, and stored at 4 °C.

Immobilization of *Bs*GGT on GA-EDA-AG was performed by following a standard protocol.^23^ Briefly, GA-EDA-AG was washed abundantly with 50 mM NaHCO_3_ buffer pH 10.0 and then filtered under reduced pressure until dryness. Then, 77 μL of *Bs*GGT (6.5 mg/mL) was solubilized in 6.4 mL of NaHCO_3_ buffer. Then, 500 mg of activated carrier was added; the suspension was allowed to stir for 6 h at room temperature. Finally, 7 mg of NaBH_4_ was added to the mixture and incubated for 30 min for imine reduction. The immobilized enzyme was then filtered, washed with deionized water, and stored at 4 °C.

Immobilization of *Bs*GGT on ReliZyme EP112/S was performed as reported in the literature.^19^ Commercial ReliZyme EP112/S was hydrated with distilled water for 1 h under mechanical stirring. Then, the carrier was washed with 1 M KH_2_PO_4_ buffer pH 8.0. Then, 77 μL of *Bs*GGT (6.5 mg/mL) was solubilized in the same buffer (6.4 mL) and the carrier (500 mg) was added to the solution. The suspension was allowed to stir for 24 h at room temperature. Finally, the immobilized enzyme was filtered under vacuum, washed abundantly with deionized water, and then resuspended in 7 mL of 50 mM NaHCO_3_ buffer pH 10.0 containing 3 M glycine, 1.5 M cysteine, or 3 M glucosamine in order to quench the unreacted epoxy groups. After 20 h, the immobilized biocatalyst was washed with deionized water and stored at 4 °C.

Immobilization of *Bs*GGT on GLX-ReliZyme was performed by following a standard protocol.^19^ Briefly, GLX-ReliZyme was washed abundantly with 50 mM NaHCO_3_ buffer pH 10.0 and then filtered under reduced pressure until dryness. Then, 77 μL of *Bs*GGT (6.5 mg/mL) was solubilized in 6.4 mL of NaHCO_3_ buffer. Then, 500 mg of carrier was added and the suspension was allowed to stir for 3 h at room temperature. Finally, 7 mg of NaBH_4_ was added to the mixture and incubated for 30 min for imine reduction. The immobilized enzyme was then filtered, washed with deionized water, and stored at 4 °C.

Immobilization of *Bs*GGT on GA-EDA-ReliZyme was performed by following a standard protocol.^19^ Briefly, GA-EDA-AG was washed abundantly with 50 mM NaHCO_3_ buffer pH 10.0 and then filtered under reduced pressure until dryness. Then, 77 μL of *Bs*GGT (6.5 mg/mL) was solubilized in 6.4 mL of NaHCO_3_ buffer and added to the activated carrier (500 mg); the suspension was allowed to stir for 6 h at room temperature. Finally, 7 mg of NaBH_4_ was added to the mixture and incubated for 30 min for imine reduction. The immobilized enzyme was then filtered, washed with deionized water, and stored at 4 °C.

Immobilization of *Bs*GGT on GlcN-ReliZyme was performed by following a standard protocol.^22^ Briefly, GlcN-ReliZyme was washed abundantly with 50 mM NaHCO_3_ buffer pH 10.0 and then filtered under reduced pressure until dryness. Then, 77 μL of *Bs*GGT (6.5 mg/mL) was solubilized in 6.4 mL of NaHCO_3_ buffer pH 10.0 and added to the activated carrier (500 mg); the suspension was allowed to stir for 3 h at room temperature. Finally, 7 mg of NaBH_4_ was added to the mixture and incubated for 30 min for imine reduction. The immobilized enzyme was then filtered, washed with deionized water, and stored at 4 °C.

#### Batch Synthesis of γ-Glutamyl-Derivatives: Analytical Scale

The synthesis of γ-glutamyl-derivatives was performed in a 2 mL reaction volume containing 100 mM l-glutamine (γ-E-donor) and 100 mM acceptors at pH 10.0 in H_2_O. The reactions were initiated by adding 0.5 UI/mL of *Bs*GGT-GLX-AG (17 UI/g) and incubated at 25 °C under magnetic stirring for 24 h. Samples were periodically withdrawn and derivatized with Sanger’s reagent before analysis by following a standard protocol.^15^ HPLC analyses were carried out by using a linear gradient of eluent A (water + 0.1% TFA) and eluent B (acetonitrile + eluent A 80:20): 0–10 min A/B 80:20 isocratic, 10–15 min A/B 70:30 linear gradient; 15–25 min A/B 70:30 isocratic; 25–30 min A/B 60:40 linear gradient; 30–35 min A/B 50:50 linear gradient; 35–40 min A/B 40:60 linear gradient; 40–60 min A/B 60:40 linear gradient; 60–70 min A/B 80:20 isocratic; flow rate: 1 mL/min; UV-detection: 356 nm; 25 °C.

#### Batch Synthesis of γ-Glutamyl-Derivatives: Semipreparative Scale and Purification

Once the endpoint for each reaction was determined (analytical scale), the reactions were scaled up (15 mL). The reactions were initiated by adding 0.5 UI/mL *Bs*GGT-GLX-AG (17 UI/g) and incubated at 25 °C under magnetic stirring for 3 h for the synthesis of γ-E-Met (**1**), γ-E-(*S*)-allyl-Cys (**2**), and ophthalmic acid (**4**) and 24 h for the synthesis of γ-E-Tau (**3**), respectively. The reactions were filtered under reduced pressure to remove the immobilized enzyme, and the solution was loaded onto a Dowex 1 × 8 ion exchange resin (200–400 mesh) in the acetate form for product purification. The elution was performed with AcOH (0.1, 0.5, 1, 2, and 2.5 M: three column volumes each). For γ-E-Tau (**3**), an additional elution step was performed with HCOOH/AcOH/water 1:2:16 as reported by Suzuki et al.^24^ Fractions containing the desired product (TLC control) were combined and freeze-dried. The structure of the isolated products was confirmed by ^1^H-NMR and ESI-MS analyses.

#### *Bs*GGT-GLX-AG Shelf-Life

The immobilized enzyme was stored at 4 °C, and its activity was measured periodically by the standard activity assay described above.

#### Flow Synthesis of γ-Glutamyl-(*S*)-Allyl-Cysteine (**2**): General Procedure

First, 330 mg of *Bs*GGT-GLX-AG (enzyme loading: 1 mg/g; activity: 17 UI/g) was packed in an Omnift glass column (6.6 mm i.d. × 100 mm length) with a final volume of 0.5 mL. A solution of (*S*)-allyl-cysteine (SAC, 100–600 mM, 1.0 mL) and a solution of l-glutamine (200–800 mM, 1.0 mL) were prepared in 100 mM carbonate buffer pH 10.0. The two solutions were mixed in a T-junction and flowed through the packed bed reactor (residence time: 2–60 min; *T* = 25–37 °C; *P* = atm-16 bar). The exiting flow stream was collected at the steady state and analyzed by HPLC (Table S1).

#### Flow Synthesis and In-Line Purification of γ-Glutamyl-(*S*)-Allyl-Cysteine (**2**)

Step I: 1.0 g of *Bs*GGT-GLX-AG (enzyme loading: 1 mg/g; activity: 17 UI/g) was packed in an Omnift glass column (6.6 mm i.d. × 100 mm length) with a final volume of 1.5 mL. A solution of (*S*)-allyl-cysteine (SAC; 600 mM, 10 mL, 6.0 mmol, 967 mg) and a solution of l-glutamine (200 mM, 10 mL, 2.0 mmol, 292 mg) were prepared in 100 mM carbonate buffer pH 10.0. The two solutions were mixed in a T-junction and flowed through the packed bed reactor with a total flow rate of 0.30 mL/min (0.15 mL/min each pump, residence time: 5 min).

Step II: product purification was performed by ion exchange chromatography using a Dowex 1 × 8 resin (200–400 mesh, acetate form). Then, 15 g of Dowex 1 × 8 in acetate form, previously conditioned with 5 volumes of 0.5 M AcOH, was packed in a Omnifit glass column (15 mm i.d. × 150 mm length) with a final volume of 20 mL and the solution exiting from the bioreactor was directed through the column for 90 min. Then, 100 mL of water was flowed through the column using a third HPLC pump at 0.5 mL/min, followed by three solutions of AcOH in water (0.5, 1.0, and 2.0 M, 60 mL each solution) at 0.3 mL/min. The exiting solution was collected in aliquots of 10 mL. The aliquots containing the final product were collected, the solvent was removed under reduced pressure, and the final product (**2**) was isolated in 42% yield (244 mg). The ^1^H-NMR spectrum was consistent with that previously reported.^18^

## Results and Discussion

### Screening of Immobilization Carriers

GGTs are heterodimeric proteins constituted by a large subunit and a small subunit. Generally, GGT-catalyzed reactions are performed at alkaline pH (8.5–11.0) in order to control and reduce the side reactions (i.e., autotranspeptidation and hydrolysis). However, microbial GGTs are unstable and subjected to subunit dissociation under strong alkaline conditions, thus leading to irreversible decline in enzyme activity.^25,26^ The hydrolase activity of *Bs*GGT used in this work was reported to reach a maximum at pH ∼ 9.0, while the maximum transpeptidation reaction occurred at pH ∼ 10.0 with a marked decrease (∼half of the initial velocity) at pH 11.0.^6^

*Bs*GGT was covalently immobilized on two different types of carriers with the goal of developing a robust *Bs*GGT-based biocatalyst for preparative applications: ReliZyme 112/S, which is a hydrophobic matrix, and agarose, which is a sharply hydrophilic carrier. Both carriers are versatile since they can be easily functionalized by exploiting the reactivity of either epoxy groups or hydroxyl groups for ReliZyme 112/S and agarose, respectively.^27^ The immobilization of *Bs*GGT on ReliZyme 112/S (Table 1, entry **1**) gave good yields in terms of immobilized protein (50–58%), whereas the activity recovery was modest (14–23%). Taking into account that the immobilization yield referred to the activity is higher than 90%, it is plausible that a partial deactivation of the enzyme under the immobilization conditions occurred. Three different quenching reagents (i.e., glycine, cysteine, and glucosamine) were tested as these molecules can differently modulate the microenvironment surrounding the enzyme, thus influencing the catalysis.^20^ However, the quenching reagent did not seem to have an impact on the activity recovery. Epoxy groups of ReliZyme 112/S were converted into aldehyde groups via ring opening and oxidation (Table 1, entry **2**). The immobilization yields (99%) and the activity recovery (41%) resulted to be higher compared to ReliZyme 112/S, as a result of a more favorable interaction of aldehyde over epoxy groups and a shorter immobilization time (3 h vs 24 h). When *Bs*GGT was immobilized on aldehyde-activated ReliZyme 112/S carrying a spacer (Table 1, entries **3** and **4**) in order to ensure a higher flexibility to the immobilized enzyme, immobilization yield and activity recovery of GA-EDA-ReliZyme 112/S (Table 1, entry **3**) and GlcN-ReliZyme (Table 1, entry **4**) were comparable but lower than GLX-ReliZyme 112/S. In the case of GlcN-ReliZyme 112/S, at a higher concentration of glucosamine (1.5 M) and alkaline pH, the activity recovery was increased by a 2.3-fold factor, in agreement with the results reported by Serra et al.^22^ (Table 1, entry **5**). We finally decided to test a hydrophilic carrier maintaining the aldehyde functionalization in order to investigate the influence of the microenvironment surrounding the enzyme. Agarose-based carriers (AG) are porous, mechanically resistant, and highly hydrophilic beads derived from a natural heteropolysaccharide. These features make this biopolymer an ideal carrier for enzyme immobilization.^28^ The immobilization of *Bs*GGT on GLX-AG (Table 1, entry **6**) gave a very high immobilization yield (>95%) and resulted in a “hyper-activated” derivative (almost 200% activity recovery). This effect might be ascribed to an enhanced interaction between the two subunits of *Bs*GGT which results in protein stabilization and increased activity, as recently reported for a β-xylosidase from *B. subtilis*.^29^ We decided to investigate also the GA-EDA-AG carrier (Table 1, entry **7**) in order to compare it to the GA-EDA-ReliZyme 112/S derivative, and also in this case, the immobilization yield was comparable (80%), but the activity recovery was higher (82% vs 9%), thus corroborating the choice of a hydrophilic carrier and a shorter immobilization time (6 h vs 24 h).

**Table 1:**
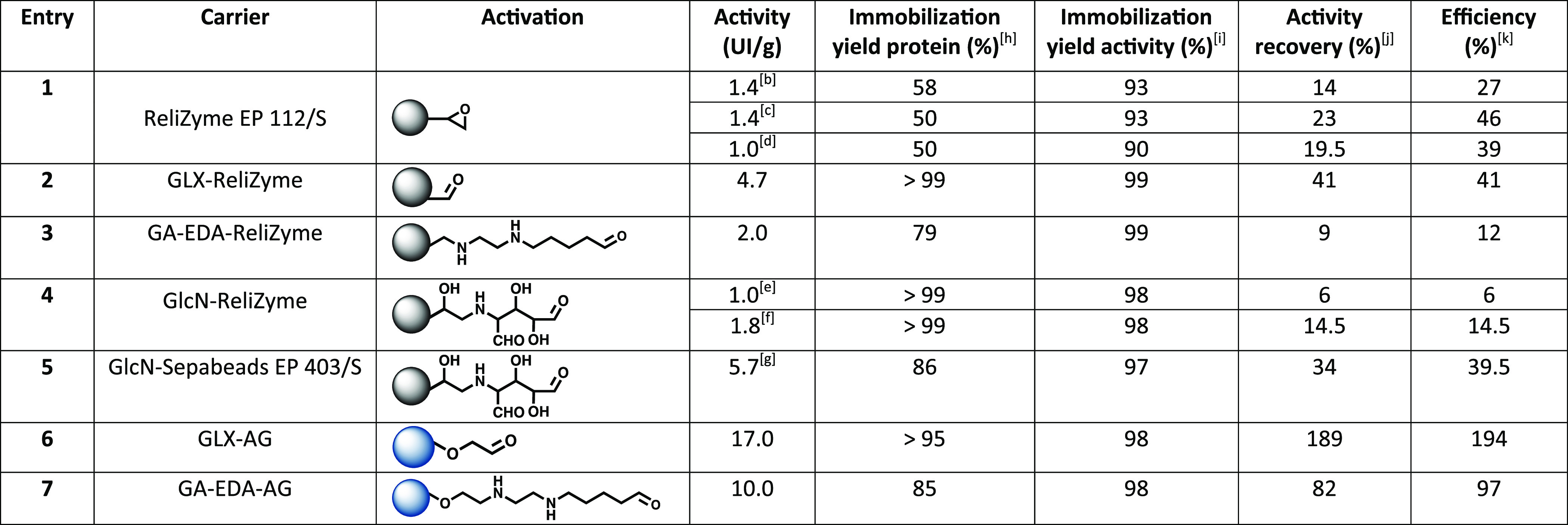
Covalent Immobilization of *Bs*GGT on ReliZyme EP 112/S- and Agarose-Based Carriers^a^

a Loading: 1 mg *Bs*GGT per g of support. AG (agarose); GLX-AG (glyoxyl-agarose); GA (glutaraldehyde); EDA (ethylenediamine); ReliZyme EP 112/S; GlcN (glucosamine).

b Immobilization yield based on protein concentration (%) was calculated based on Bradford assay as follows: (immobilized protein/loaded protein) × 100.

c Immobilization yield based on activity (%) was calculated based on the spectrophotometric standard activity assay as follows: (immobilized activity/loaded activity) × 100.

d Activity recovery (%) was calculated as follows: (observed activity of the immobilized enzyme/starting activity) × 100.

e Efficiency (%) was calculated as follows: (observed activity of the immobilized enzyme/immobilized activity) × 100.

f Quenching with 3 M glycine.

g Quenching with 1.5 M cysteine.

h Quenching with 3 M glucosamine.

i Carrier activation with 1 M glucosamine at pH 7.5.

j Carrier activation with 1 M glucosamine at pH 10.0.

k Carrier activation with 1.5 M glucosamine at pH 10.0.^22^

Based on these results, GLX-AG was selected as the carrier for the immobilization of *Bs*GGT (high immobilization yield and activity). A further strength point of *Bs*GGT-GLX-AG was its shelf-life: this immobilized preparation retained 90% of its starting activity after 10 months at 4 °C (Figure S1). *Bs*GGT-GLX-AG was used, indeed, for the batch and flow synthesis of the γ-E-derivatives reported herein.

### Batch Synthesis of γ-E-Derivatives (**1**–**4**)

The biotransformations were first performed on an analytical scale in order to determine the endpoints allowing the highest conversion of the target γ-E-derivatives and limiting the formation of byproducts. All the reactions reached the highest conversion after 3 h, with the exception of γ-E-Tau (24 h).

Then, to demonstrate the versatility of the biocatalytic application of *Bs*GGT-GLX-AG, the biocatalyst was used in the synthesis of different γ-E-derivatives, i.e., γ-E-Met (**1**), γ-E-(*S*)-allyl-Cys (**2**), γ-E-Tau (**3**), and ophthalmic acid (**4**) at a semipreparative scale (15 mL). *Bs*GGT-GLX-AG (7.5 IU) was added to 100 mM acceptors in the presence of 100 mM l-glutamine (l-Gln) (γ-E-donor) in water at pH 10.0. After 3 h (24 h for γ-E-Tau) at 25 °C, the products were isolated by preparative ion exchange chromatography in moderate yields (20–40%) and good purity (>80%) as shown in Table 2.

**Table 2:** Batch Synthesis of γ-E-Derivatives Catalyzed by *Bs*GGT-GLX-AG

γ-E-derivative	reaction time (h)	conversion (%)^a^	product (mg)^b^	purity (%)	yield (%)^c^
γ-E-Met (**1**)	3	30	117	>99^d^	28
γ-E-(*S*)-allyl-Cys (**2**)	3	27	100	88^d^	20
γ-E-Tau (**3**)	24	44	188	90^e^	40
Ophthalmic acid (**4**)	3	20	97	84^d^	19

a Determined by HPLC using calibration curves of the product.

b After lyophilization of fractions collected from ion exchange column chromatography.

c Referred to the pure product.

d On the molar basis, estimated by NMR; the main impurity was γ-E-Gln which partially co-eluted with the product during ion-exchange column chromatography.

e Estimated by HPLC.

The main byproducts found in the crudes at the reaction endpoint (HPLC analysis) were glutamic acid (6–13 mol %), (γ-E)_2_-derivatives of the acceptor substrate (4–10 mol %), and γ-E-Gln (3–6 mol %) (Figures S2–S5). γ-E-Gln partially co-eluted with the target products and was detected in the ^1^H NMR spectra of compounds **2**–**4**. Through integration of the signals attributable to the product and to γ-E-Gln, it was possible to estimate their relative molar amount in the sample, allowing for the calculation of the reaction yields as reported in Table 2. The presence of (γ-E)_2_-(*S*)-allyl-Cys and (γ-E)_2_-Tau in the crudes of the respective reactions was also confirmed by their isolation through ion exchange column chromatography.

The enzyme-catalyzed production of γ-E-Tau (**3**) was much slower with respect to the other reported examples, and an almost complete consumption of the donor l-Gln was observed only after 24 h. Due to the high acidity of γ-E-Tau (**3**), its elution from the resin required a concentrated solution of acetic and formic acid. The HPLC chromatogram of the obtained sample revealed traces of unidentified byproducts. Based on their retention times, these peaks were tentatively assigned to poly-γ-E derivatives of both Tau and l-Gln (Figure S5). The formation of small amounts of poly-γ-E derivatives carrying several γ-glutamyl moieties bound to a single acceptor molecule is a recurrent trait of *B. subtilis* GGT-catalyzed reactions.^8,14^

### Flow Synthesis of γ-E-(*S*)-Allyl-Cysteine (2): Study of Reaction Parameters

**Scheme 2:**
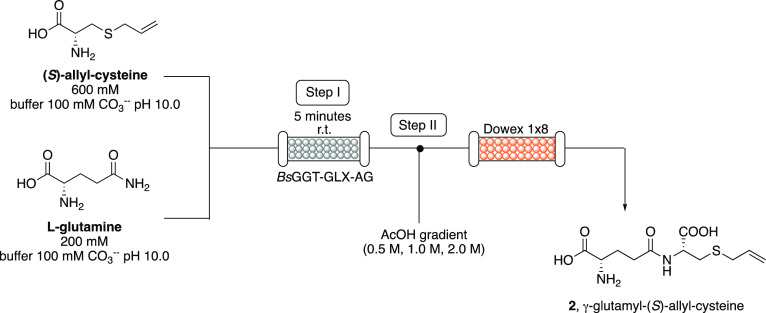
Reactor Configuration of the Biocatalyzed Flow Synthesis (Step I) and In-Line Purification (Step II) of γ-Glutamyl-(*S*)-Allyl-Cysteine (**2**)

To intensify the production of the γ-E-peptides, the immobilized biocatalyst was integrated in a continuous flow packed bed reactor (PBR). For the optimization of the reaction parameters, we focused on the reaction between l-Gln and (*S*)-allyl-cysteine (SAC) to obtain compound **2**. A bioreactor was prepared by packing the immobilized biocatalyst (0.33 g) in a glass column (final volume 0.5 mL). The solutions of l-Gln and SAC (1 mL each) in sodium carbonate buffer (0.1 M, pH 10.0) were mixed using a T piece and directed into the PBR (Scheme 2, step I). First, the influence of the residence time on the reaction outcome was investigated (Table S1, entries **2–12**). All the samples were collected at the steady state and analyzed by HPLC. The best results in terms of conversion into the desired product **2** and byproduct formation were obtained after 5 min of residence time. Lower residence time (i.e., 2 min, Table S1, entry **8**) did not allow a satisfactory conversion to be achieved, whereas longer residence times (i.e., 15, 30, and 60 min, Table S1, entries **1–5**) led to the formation of a relevant amount of glutamic acid (Glu). The increase of the temperature to 30 and 37 °C or the use of a higher pressure (8 bar) did not influence the reaction outcome. Then, the influence of the concentration and stoichiometry over the reaction profile was evaluated (Table S1). When the concentration of substrates was increased from 100 to 200 mM, a slightly lower percentage of compound **2** was formed (Table S1, entry **12**); however, an increase in the residence time to 15 min resulted in a profile similar to the batch reaction but with a considerable shorter time (15 min instead of 3 h) (Table S1, entry **13**). The change in the stoichiometric ratio between l-Gln and SAC favoring l-Gln (i.e., 2:1 and 3:1) did not result in a significant amelioration of the impurity profile, the conversion to γ-glutamyl-(*S*)-allyl-cysteine (γ-E-SAC) being reduced by more than 50% (Table S1, entries **14**, **15**). On the other hand, an increased concentration of SAC resulted in a higher formation of the desired product (Table S1, entries **20–25**); the conditions reported in Table S1, entry **23** (i.e., residence time = 5 min, *T* = 25 °C, [Gln] = 100 mM, [SAC] = 300 mM, *P* = atm) led to the formation of compound **2** in 42.8% conversion (determined by HPLC). This result was consistent with the conversion obtained in the batch synthesis of γ-E-SAC under the same conditions (Table S1, entry batch 2). It is worth noting that the immobilized biocatalyst was routinely checked (every 5 flow reactions) by collecting a sample from the bioreactor and performing a batch activity assay. *Bs*GGT-GLX-AG showed an excellent stability under continuous work, even after increasing the temperature at 37 °C. As a mild decrease of the activity was observed after pressurization, reactions were preferably performed under ambient pressure.

### Flow Synthesis of γ-E-(*S*)-Allyl-Cysteine (2) and In-Line Purification

Under optimized conditions (i.e., residence time = 5 min, *T* = 25 °C, [Gln] = 100 mM, [SAC] = 300 mM, *P* = atm, Table S1, entry **23**), the biotransformation was scaled-up (injection volume: 10 mL of each substrate solution) and an in-line purification procedure was developed by using an ionic exchange resin (Scheme 2, step II). The flow stream exiting from the bioreactor was directed into a column packed with previously conditioned Dowex 1 × 8 in the acetate form. Then, product **2** was eluted using a gradient of AcOH in H_2_O (0.5 M, 1.0 M, 2.0 M), obtaining a 45% conversion and a 42% isolated yield (244 mg).

At a similar degree of conversion (batch 1 vs flow 1 and batch 2 vs flow 2), specific productivity (SP) under flow conditions was about 1.5–1.6 fold higher than that obtained in batch synthesis (Table 3).

**Table 3:** Specific Productivity of Batch and Flow Systems

reaction	[Gln]:[SAC] (mM)	time (min)	*T* (°C)	*P* (bar)	γ-E-SAC (%)	Glu (%)	specific productivity^a^ (μmol/min*g)
batch 1	100:100	180	25	1	30	9	5.7
batch 2	100:300	180	25	1	48	13	9.1
flow 1	100:100	5	25	1	31	24	9.2
flow 2	100:300	5	25	1	45	12	13.7

a Specific productivity (SP) for flow biotransformation (SP_f_) was calculated from the concentration of the formed product ([*P*] expressed as μmol/mL), the flow rate of the liquid phase (*f* expressed as mL/min), and the mass of the immobilized enzyme (*m* expressed as g), according to the following equation: SP_f_ = [*P*] × *f*/*m* (μmol/min*g).^30,31^

In this study, the synthesis of biologically active γ-E-peptides such as γ-E-Met (**1**), γ-E-(*S*)-allyl-Cys (**2**), γ-E-Tau (**3**), and ophthalmic acid (**4**) catalyzed by covalently immobilized *Bs*GGT was set-up in a fully aqueous medium. These compounds were obtained in moderate isolated yields (19–40%, 5–10 g/L) when the reaction was performed in batch synthesis. When the GGT-catalyzed reaction was integrated in a flow system combined with an in-line downstream processing for γ-E-*S*-allyl-Cys (**2**), a significant reduction of the reaction time from 180 min (in batch) to 5 min (in flow) and a slight increase in specific productivity (13.7 μmol/min*g vs 9.1 μmol/min*g) were achieved. Nevertheless, the control of side reactions in the flow system resulted to be more challenging than expected. In fact, the improvement in reaction time and specific productivity was not accompanied by a paralleled improvement of the impurity profile.

## Supporting Information Available

(Figure S1) Shelf-life of *Bs*GGT-GLX-AG under storage (4 °C); (Figure S2) Preparative synthesis of γ-E-methionine (**1**) at the 3 h endpoint; (Figure S3) Preparative synthesis of γ-E-(*S*)-allyl-cysteine (**2**) at the 3 h endpoint; (Figure S4) Preparative synthesis of γ-E-taurine (**3**) at the 24 h endpoint; (Figure S5) Preparative synthesis of ophthalmic acid (**4**) at the 3 h endpoint; and (Table S1) In-flow synthesis of γ-E-(*S*)-allyl-cysteine (**2**): study of reaction parameters (PDF)
