# Supplementary material for: From Batch to Continuous Flow Bioprocessing: Use of an Immobilized γ-Glutamyl Transferase from B. subtilis for the Synthesis of Biologically Active Peptide Derivatives
Source: J Agric Food Chem. 2022 Sep 23;70(42):13692–9. doi: 10.1021/acs.jafc.2c03702 (PMC9614966; doi:10.1021/acs.jafc.2c03702)
Supplement: Supplementary file 1 — jf2c03702_si_001.pdf [file jf2c03702_si_001.pdf]

SUPPORTING INFORMATION

to the article

From Batch to Continuous Flow Bioprocessing: Use of an Immobilized γ-Glutamyl Transferase from *B. subtilis* for the Synthesis of Biologically Active Peptide Derivatives

Marina S. Robescu[a], Francesca Annunziata[b], Valeria Somma[c], Cinzia Calvio[d], Carlo F. Morelli[c], Giovanna Speranza[c], Lucia Tamborini[b], Daniela Ubiali[a], Andrea Pinto[e]*, Teodora Bavaro[a]*

[a] Department of Drug Sciences, University of Pavia, viale Taramelli 12, Pavia, Italy

[b] Department of Pharmaceutical Sciences, University of Milano, via Mangiagalli 25, Milano, Italy

[c] Department of Chemistry, University of Milano, via Golgi 19, Milano, Italy

[d] Department of Biology and Biotechnology "L. Spallanzani", University of Pavia, via Ferrata 1, Pavia, Italy

[e] Department of Food, Environmental and Nutritional Sciences, University of Milano, via Celoria 2, Milano, Italy

*Corresponding authors: teodora.bavaro@unipv.it

andrea.pinto@unimi.it

Table of contents

Supplementary Figures

Figure S1. Shelf-life of *Bs*GGT-GLX-AG under storage (4 °C) 1

Figure S2. Preparative synthesis of γ-E-methionine (**1**) at the 3 hours endpoint 2

Figure S3. Preparative synthesis of γ-E-(*S*)-allyl-cysteine (**2**) at the 3 hours endpoint 2

Figure S4. Preparative synthesis of γ-E-taurine (**3**) at the 24 hours endpoint 3

Figure S5. Preparative synthesis of ophthalmic acid (**4**) at the 3 hours endpoint 3

Supplementary Tables

Table S1. In-flow synthesis of γ-E-(*S*)-allyl-Cys (**2**): study of reaction parameters 4

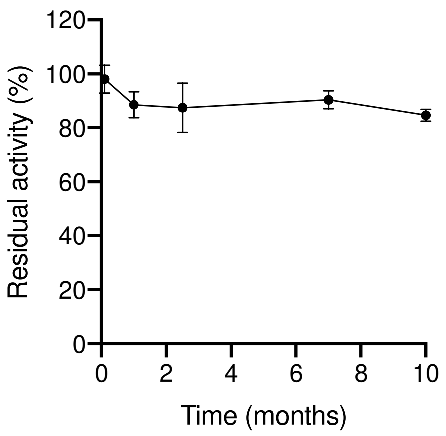

Figure S1. Shelf-life of *Bs*GGT-GLX-AG under storage (4 °C).

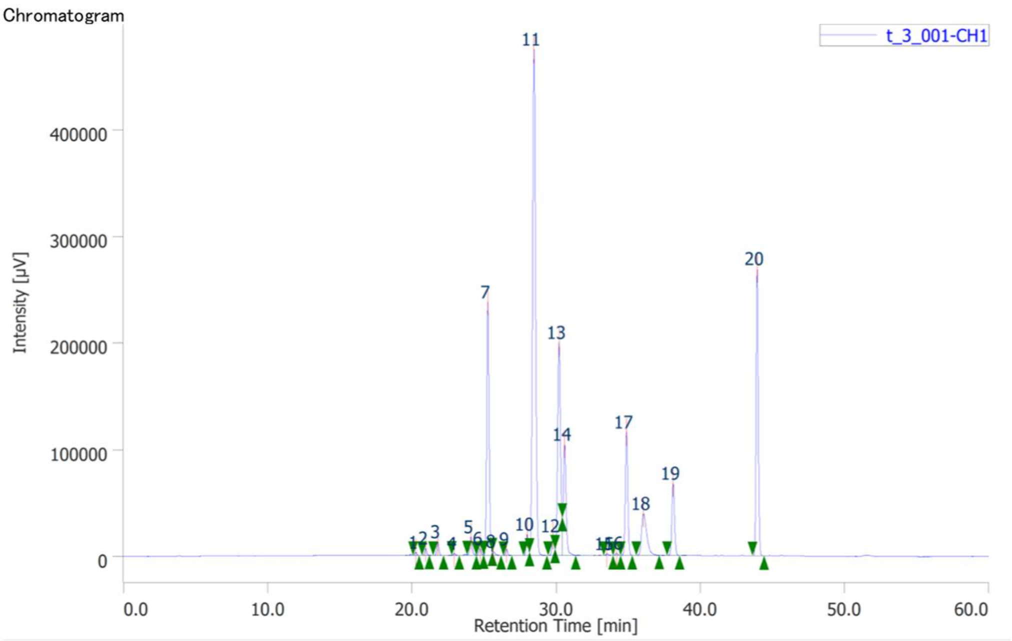

Figure S2. Preparative synthesis of γ-E-methionine (**1**) at the 3 hours endpoint. 1= γ-E_3-Gln; 2= γ-E_2-Gln; 3= γ-E-Gln; 5= Gln; 7= serine (internal standard); 11= excess of Sanger's reagent; 12 = glutamic acid; 13= ammonia; 14= derivatization artifact; 17= γ-E-methionine (**1**); 18 & 19= derivatization artifacts; 20= methionine.

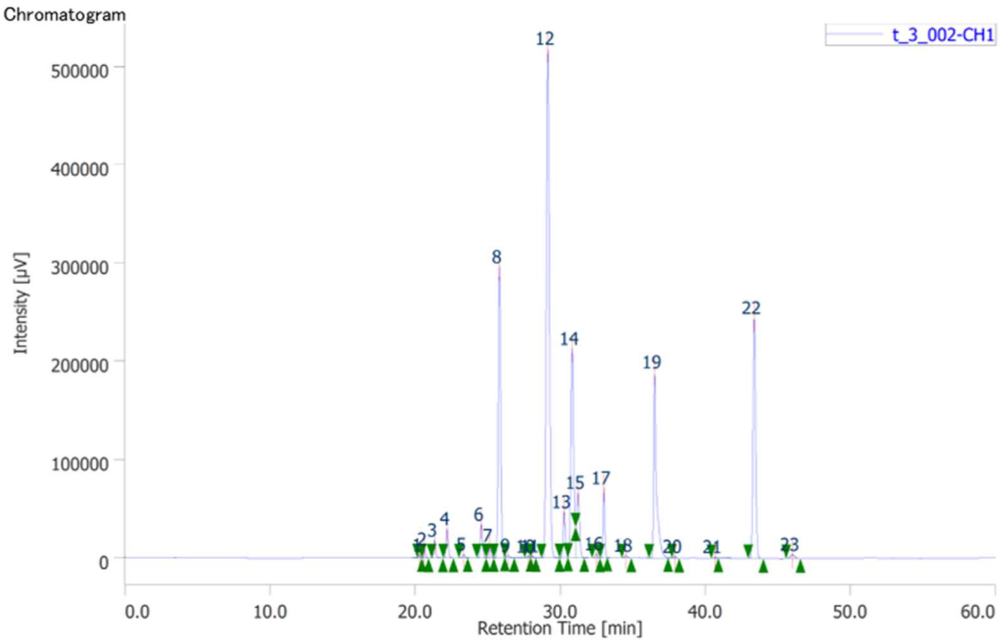

Figure S3. Preparative synthesis of γ-E-(*S*)-allyl-cysteine (**2**) at the 3 hours endpoint. 2= γ-E_3-Gln; 3= γ-E_2-Gln; 4= γ-E-Gln; 6= Gln; 8= serine (internal standard); 12= excess of Sanger's reagent; 13= glutamic acid; 14= ammonia; 15= derivatization artifact; 17= γ-E_2-(S)-allyl-cysteine; 19= γ-E-(*S*)-allyl-cysteine (**2**) (overlaps with the peak of a derivatization artifact; the conversion of **2** was corrected taking into account the peak area of the derivatization artifact); 22= (*S*)-allyl-cysteine.

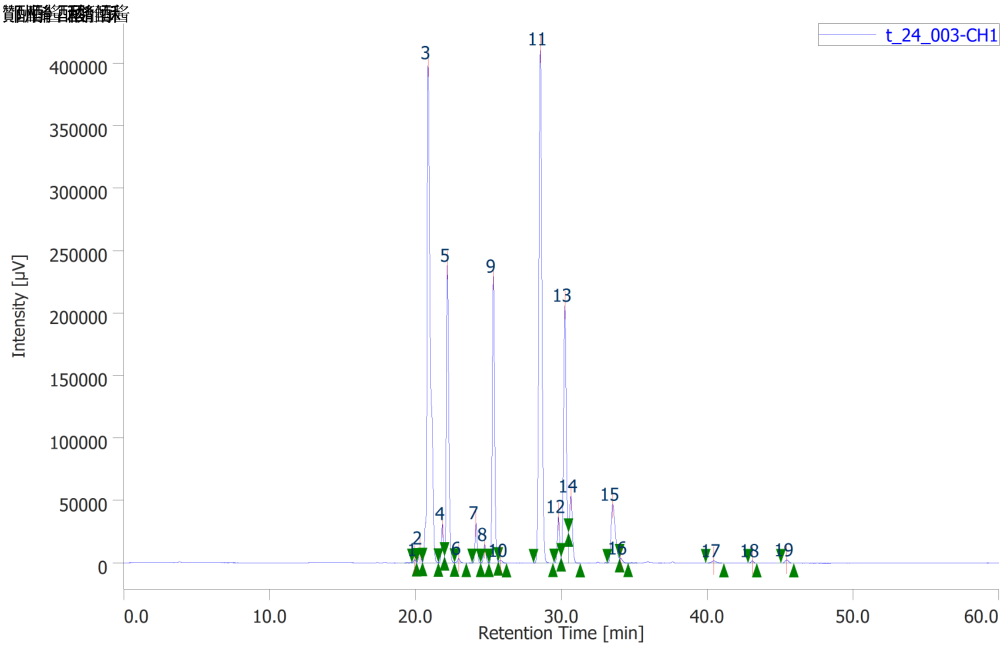

Figure S4. Preparative synthesis of γ-E-taurine (**3**) at the 24 hours endpoint. 3= taurine; 4= γ-E-Gln; 5=γ-E-taurine (**4**); 7= Gln; 9= serine (internal standard); 11= excess of Sanger's reagent; 12= glutamic acid; 13= ammonia; 14 and 15= derivatization artifacts.

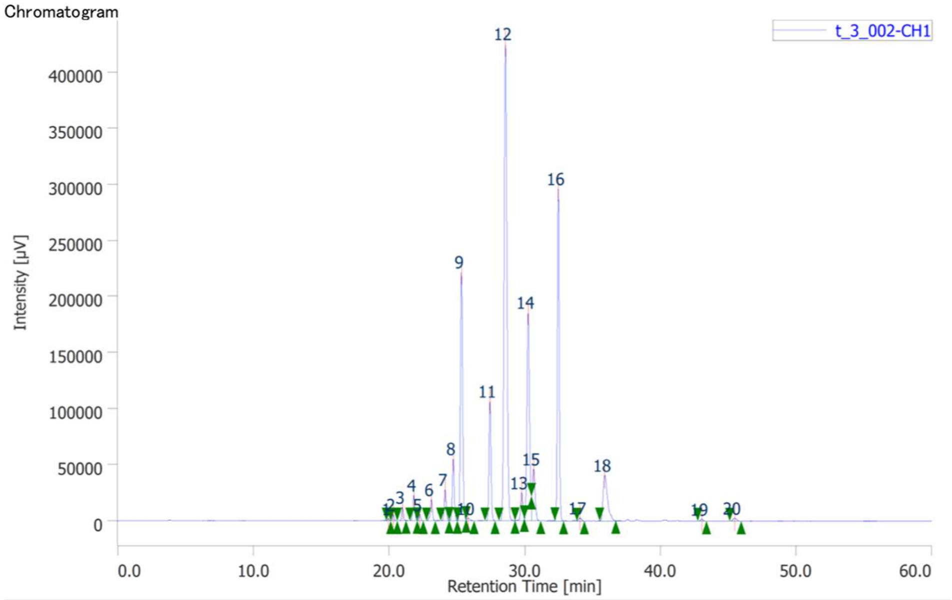

Figure S5. Preparative synthesis of ophthalmic acid (**4**) at the 3 hours endpoint. 2= γ-E_3-Gln; 3= γ-E_2-Gln; 4= γ-E-Gln; 6= putative γ-E_3-AbuGly; 7= Gln; 8= putative γ-E_2-AbuGly; 9= serine (internal standard); 11= ophthalmic acid (γ-E-AbuGly, **3**); 12= excess of Sanger's reagent; 13= glutamic acid; 14= ammonia; 15= derivatization artifact; 16= AbuGly; 18= derivatization artifact.

Table S1. In-flow synthesis of γ-E-(*S*)-allyl-Cys (**2**): study of reaction parameters.

Entry	[L-Gln][a] (mM)	[SAC][a] (mM)	Res. time (min)	T (°C)	P (bar)	Gln (%)	γ-E-SAC (2) (%)	Glu (%)	γ-EE-SAC (%)	γ-EEE-SAC (%)	γ-E-Gln (%)	γ-EE-Gln (%)	γ-EEE-Gln (%)	γ-E-Glu (%)
**Batch 1**	100	100	180	25	atm	6.4	30.2	9.1	12.6	0.7	5.3	2.5	1.0	2.4
**Batch 2**	100	300	180	25	atm	4.2	48.4	12.7	8.9	2.2	2.8	0.6	0	1.6
**1**	100	100	15	25	atm	1.4	18.5	58.0	4.6	0	1.4	0	0	5.0
**2**	100	100	30	25	atm	0	11.0	82.2	0	0	0	0	0	3.4
**3**	100	100	60	25	atm	0	4.6	87.4	0	0	2.2	0	0	1.8
**4**	100	100	30	30	atm	0	9.5	78.9	0	0	2.4	0	0	3.4
**5**	100	100	15	30	atm	2.4	28.1	32.8	6.9	2.3	1.8	1.0	0	4.8
**6**	100	100	5	30	atm	10.5	29.2	18.4	8.3	1.5	5.4	1.8	0	2.4
**7**	100	100	5	25	atm	15.1	30.5	11.1	6.9	1.7	7.5	1.8	0	2.1
**8**	100	100	2	25	atm	38.1	19.8	4.5	3.6	0.9	11.2	1.8	0	0
**9**	100	100	5	37	atm	11.2	26.8	21.4	7.6	1.4	5.3	1.7	0	2.7
**10**	100	100	15	37	atm	1.7	21.3	47.4	4.6	1.7	0.8	1.5	0	4.7
**11**	100	100	5	25	8	8.7	32.5	24.1	8.8	0	5.6	0	0	3.0
**12**	200	200	5	25	atm	22.1	22.5	10.3	5.4	1.6	9.9	2.4	0	1.4
**13**	200	200	15	25	atm	3.6	30.4	31.3	8.4	0	3.4	1.2	0.5	1.5
**14**	100	50	5	25	atm	13.2	15.2	18.8	5.5	0	9.4	3.9	1.2	3.2
**15**	150	50	5	25	atm	22	9.9	8.3	3.3	0.7	14.1	5.1	1.2	1.4
**16**	200	200	5	25	8	1	24.2	15.2	7.2	0	9.1	3.0	0	2.5
**17**	200	200	5	25	16	17.6	26.2	12.4	7.7	0	8.3	2.7	0	1.9
**18**	400	200	5	25	8	27	15.3	6.5	4.1	0	13.3	3.8	0.8	0.8
**19**	400	200	5	25	atm	23.7	17.3	8.4	4.2	0	12.2	3.8	0.9	1.4
**20**	100	200	5	25	atm	8.3	35.0	17.2	9.1	1.0	4.5	1.4	0.3	2.0
**21**	100	200	15	25	atm	1.9	35.0	33.6	8.0	0.9	1.4	0.4	0	3.4
**22**	100	200	5	25	8	6.2	32.9	20.5	8.2	1.1	6.1	1.1	0	2.4
**23**	100	300	5	25	atm	12.9	45.2	11.6	6.5	1.1	4.7	0.8	0	1.1
**24**	100	300	15	25	atm	1.7	40.6	31.6	7.4	1.4	1.2	0	0	2.3
**25**	100	300	5	25	8	9.6	38.0	15.3	8.5	1.2	4.6	1.3	0	1.6

[a] [L-Gln] and [SAC] resulting after mixing in the T-piece.
